# Preparation and Application of Biocontrol Formulation of Housefly—Entomopathogenic Fungus—*Metarhizium brunneum*

**DOI:** 10.3390/vetsci12040308

**Published:** 2025-03-28

**Authors:** Chengyu Ma, Luyao Hao, Zhengyi Li, Yuan Ma, Rui Wang

**Affiliations:** 1 College of Veterinary Medicine, Inner Mongolia Agricultural University, Hohhot 010018, China; 2 Key Laboratory of Clinical Diagnosis and Treatment of Animal Diseases, Ministry of Agriculture, National Animal Medicine Experimental Teaching Center, Hohhot 010018, China

**Keywords:** biological control, combination preparation, deltamethrin, entomopathogenic fungus, housefly, *Metarhizium brunneum*

## Abstract

Utilizing entomopathogenic fungi as a biological control method for parasitic houseflies in livestock presents a promising and innovative alternative. This study aimed to evaluate the control efficacy of a lyophilized *Metarhizium brunneum* preparation, both alone and in combination with the insecticide low-dose deltamethrin, to control houseflies in Hu sheep on a farm based in Gansu Province, northwestern China. The experimental design included four groups: *M. brunneum* wettable powder (WP) treatment group, *M. brunneum* + deltamethrin combination WP treatment group, deltamethrin group, and a control group. By observing the mortality rate of houseflies at 1, 3, 5, and 7 days post-treatment, the data were analyzed, and control efficacy was calculated to evaluate the effectiveness of each treatment group on houseflies at different time points. On day 7, the combination of WP with deltamethrin resulted in 79.2% control of housefly larvae and pupae and 85.42% control of adult houseflies, respectively, surpassing the efficacy observed in the deltamethrin-only group. The incorporation of low-dose insecticides into the fungal treatment increased the mortality rate by 17.1% compared to the use of *M. brunneum* alone, significantly reducing the overall pesticide usage while enhancing the efficacy of biological control.

## Simple Summary

Houseflies in livestock-raising areas can create numerous issues. They not only spread diseases but also bother the animals. Researchers have long been seeking more effective methods to manage them. In this research, a fungal product named *Metarhizium brunneum* (Petch) was mixed with a low-dose insecticide, deltamethrin, to test their ability to control houseflies around Hu sheep (*Ovis aries*) on a Gansu Province, northwestern China-based farm. After seven days of application, this combination demonstrated remarkable control over housefly larvae, pupae, and adults, performing far better than deltamethrin used alone. This study indicates that this combination is an efficient approach to controlling houseflies in livestock environments, cutting down pesticide usage, and bringing benefits to both farming and the environment.

## 1. Introduction

The housefly, *Musca domestica* L. (*Diptera*: Muscidae), is one of the global synanthropic pests [1]. Its population constitutes a significant proportion of the total fly population [2], leading to substantial economic losses worldwide every year. This is primarily due to the large number of pathogens it carries. In the United States alone, houseflies account for USD 500 million to USD 1 billion annually in insecticide costs and total economic losses, respectively [3]. Houseflies acquire microbes on their surfaces either through contact with or by directly feeding on refuse, animal waste, wounds, and exudate [4,5]. This makes them carriers of a wide range of pathogenic bacteria, facilitating the transmission of disease between livestock and poultry [5,6]. Additionally, the bites and nuisance caused by houseflies can negatively impact the growth, development, performance, and feed utilization of livestock, resulting in considerable economic losses for the farming industry [7]. As a result, controlling housefly populations has become a critical issue for the agricultural sector.

Traditionally, chemical insecticides, such as pyrethroids, carbamates, organophosphates, organochlorines, and neonicotinoids, have been widely used to control housefly populations [7]. However, the improper use of these insecticides has led to a range of issues, including insecticide resistance, pesticide residues, and environmental pollution [8,9]. However, the limitations associated with chemical insecticides, such as resistance development and environmental pollution, underscore the need for environmentally safe alternatives [10,11]. Among these alternatives, the use of entomopathogenic fungi has emerged as a promising approach to reducing reliance on chemical pesticides. The use of entomopathogenic fungi for the biological control of insect pests offers a promising, safe, and effective alternative, as demonstrated by numerous successful applications worldwide [12,13,14,15]. *M. brunneum* is a generalist fungus known for its virulence against a variety of arthropod hosts, including mites, beetles, flies, and lepidopterans [16,17,18,19]. Specifically, previous studies have confirmed its efficacy in controlling houseflies through cuticular penetration and toxin production, making it a promising biocontrol agent for this synanthropic pest [20].

The mode of action of *M. brunneum* involves the fungus infecting the host insect, penetrating the cuticle, and proliferating within the host’s body, ultimately leading to the death of the insect [13,17,21]. The fungus produces secondary metabolites that can have insecticidal effects [22]. Furthermore, *M. brunneum* has been demonstrated to have beneficial effects on plant growth and resistance, making it a promising tool for integrated pest management [23,24].

Although entomopathogenic fungi show strong potential in biological control, their prolonged duration of action may limit their immediate effectiveness in practice. Recent studies have shown that combining chemical insecticides with entomopathogenic fungi tends to be more effective in practice than either method alone. For example, a study by Yadav et al. highlighted the compatibility of *M. brunneum* with certain insecticides, suggesting that these combinations can enhance the management of mealybug (*Maconellicoccus hirsutus*) populations without compromising the efficacy of the fungal biocontrol agent [25]. This compatibility is crucial, as it allows for the simultaneous application of biological and chemical controls, potentially leading to improved pest suppression and reduced reliance on chemical insecticides alone. Behle et al. reported that experimental preparations of *M. brunneum* were significantly effective in controlling Japanese beetle (*Popillia japonica*) larvae, indicating that the fungus can serve as a potent biological control agent when used in conjunction with chemical insecticides [26].

While biological control has shown great potential in managing parasites, reports on the synergistic effects of simultaneous application of chemicals and entomopathogenic fungi remain limited. Developing practical and cost-effective fungal formulations is crucial for successfully deploying these biocontrol agents. This study aims to prepare WP using vacuum freeze-drying and to combine it with deltamethrin to develop combination formulations. These formulations are intended to provide new solutions for the biological control of ectoparasitic diseases in livestock.

## 2. Materials and Methods

### 2.1. Fungal Strain and Tested Insecticides

One chemical insecticide was tested: deltamethrin (≥98% purity; Beijing Mreda Technology Co., Ltd., Beijing, China), using WP of lyophilized *M. brunneum* conidia and WP of lyophilized *M. brunneum* conidia–deltamethrin.

*M. brunneum* (Mb) strain KVL04-57 was isolated from an infected larva of *Cydia pomonella* (*Lepidoptera*: Tortricidae) in Austria (exact isolation origin as the active ingredient of the commercial product *Met52*, Novozymes, Salem, VA, USA). This isolate was stored at −80 °C in the laboratory of Parasites, Inner Mongolia Agricultural University, Hohhot, Inner Mongolia, China.

### 2.2. Tested Animals

The houseflies used in this experiment were harvested from captures around a lake sheep farm in Wuwei, Gansu Province, northwestern China, and were bred in the laboratory.

Adult houseflies were captured using insect nets around a Hu sheep farm in Wuwei City, Gansu Province, northwestern China, and transported to the laboratory. They were reared in rectangular plastic containers (13.2 × 8.1 × 6.3 cm). Two 6 cm Petri dishes were placed inside: one containing cotton balls soaked in a solution of brown sugar, whole milk powder, and water (5:1:50 ratio) as food, replaced daily to ensure fresh nutrition; the other filled with wheat bran (70% moisture) as an oviposition substrate. Containers were incubated at 26 ± 1 °C, 70 ± 10% relative humidity, and a 12:12 h light/dark cycle. Daily observations were conducted, and oviposition substrates were replaced. Eggs were transferred to 500 mL plastic containers with larval diet (wheat bran/cornmeal/whole milk powder = 5:2:3, 70% moisture, 5 cm depth) and incubated at 29 °C until pupation. Pupae were rinsed with water, surface-sterilized in 0.1% potassium permanganate solution for 2 min, and transferred to 500 mL containers (100 pupae per container). The lids were punctured with more than 100 holes using insect pins to facilitate airflow. Emerged adults served as stock for continuous laboratory rearing.

### 2.3. Experimental Assay

#### 2.3.1. Assay A Preparation of Fungus WP

##### Procedure of Freeze-Dried *M. brunneum* Chlamydospore Powder

**Figure 1:**
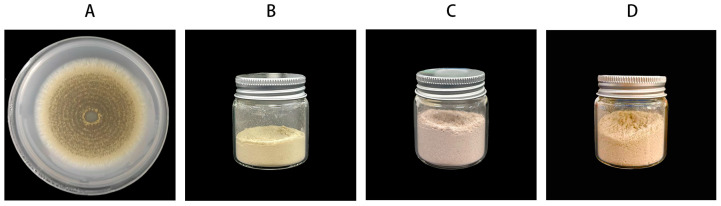
Preparation of the combination formulation. (**A**) *M. brunneum* in the potato dextrose agar medium; (**B**) *M. brunneum* chlamydospore lyophilized powder; (**C**) *M. brunneum* WP; (**D**) *M. brunneum*–deltamethrin WP.

Firstly, *M. brunneum* (Mb) strain KVL04-57 was inoculated onto potato dextrose agar (PDA) and incubated at 28 °C for 7 days (Figure 1A). The PDA medium containing pure fungi cultures was transferred to autoclaved corn kernels and incubated at 28 °C for 21 days [27]. Afterward, a spore suspension was prepared by adding a rinse solution, and the spore concentration was determined using a hemocytometer. A freeze-drying protectant was added to the spore suspension at a 1:1 ratio, and the mixture was cooled at −80 °C for 2 h. The mixture was then subjected to vacuum freeze-drying to obtain the freeze-dried fungal powder. The spore concentration in the freeze-dried fungal powder was 2.23 × 10^8^ spores/g and stored in sealed packs at 4 °C (Figure 1B).

##### Optimization of WP Excipients

Experimental and control groups were established to evaluate the effectiveness of UV protectants (skim milk, sodium ascorbate, and fluorescein sodium) in WP formulations. The test formulations comprised 20% fungal powder, 1% wetting agent, 3% dispersant, and 5% UV protectant, with diatomaceous earth used to make up to 100%. In the control group, the UV protectant was substituted entirely with diatomaceous earth. The formulated powders were diluted to a concentration of 100 *Metarhizium* spores per 10 μL, and then spread onto PDA medium plates. These plates were exposed to UV light for durations of 20 s, 40 s, and 60 s at a fixed distance from the light source, followed by incubation at 25 °C for 3 days. Germination rates were monitored periodically to determine the optimal UV protectant. Based on the selected UV protectant, further experiments were conducted to optimize its concentration, testing proportions of 1%, 2%, 3%, 4%, and 5%, using similar methodologies.

##### Preparation of *M. brunneum* WP

Through experimentation, it was determined that when the freeze-dried fungal powder ratio was 20%, the ultraviolet protectant sodium fluorescein ratio was 3%, the wetting agent ratio was 3%, and the dispersant ratio was 1%, the prepared WP exhibited the best performance in terms of solubility, stability, and other properties. To ensure that the preparation process complied with the relevant regulations of the *Pharmacopoeia of the People’s Republic of China* (2015 edition), the formulation was mixed strictly according to the pharmacopoeia guidelines to ensure the uniformity and stability of the final preparation. Subsequently, the prepared *M. brunneum* WP was subjected to comprehensive quality evaluation to ensure it met the quality standards and was ready for subsequent use.

##### Preparation of *M. brunneum*–Deltamethrin WP

Deltamethrin was added to the prepared WP of *M. brunneum* based on the housefly-specific LC_50_ value. After careful weighing, deltamethrin was thoroughly mixed to uniform formulation.

##### Preparation of Deltamethrin WP

The lethal dose of deltamethrin for houseflies was used to replace the *M. brunneum* component in the WP formulation, aiming to achieve a similar effect while ensuring lethal toxicity.

#### 2.3.2. Assay B Field Trial

One sheep pen on the farm was selected, and the animals were transferred to other sheds or pens. The sheep manure from the selected shed was collected and piled into a 1 m × 1 m square with a height of 30 cm. The manure pile was then left to ferment for 45 days before being used. Fecal samples for the experiment were obtained from the solid manure composting fermenter within the farm. The manure was first screened and filtered through a 20-mesh sieve to separate the housefly larvae and pupae. Each experimental unit consisted of a single manure pile per treatment. In each unit, 50 larvae and 50 pupae were introduced, and the dung heap was covered with a mesh net to ensure ventilation while maintaining the presence of the larvae and pupae within the netted area. After the application of treatments, each manure pile was covered with a mesh net to prevent the larvae from escaping. Four treatment groups were established, with the following dosages applied by spraying evenly onto the surface of the dung treatment groups: Group 1 (G1) received *M. brunneum* WP at a rate of 66.7 mL (ensuring a concentration of *M. brunneum* conidia greater than 1 × 10^6^ spores/mL); G2, fungi–deltamethrin at a rate of 66.7 mL; G3, deltamethrin at a rate of 66.7 mL (17.15 mg/L); G4 was given 66.7 mL of water (control). Each treatment was replicated 3 times. The number of dead housefly larvae, pupae, and surviving adults was recorded on the 1st, 3rd, 5th, and 7th days. Data were analyzed to calculate the control effect, which was applied to assess the efficacy of each treatment at different time points.

In a second experiment, the same treatment groups and spraying methods were applied. Thirty adult houseflies, temporarily anesthetized with ether, were placed in each treatment unit. The manure piles in each treatment group were covered with a mesh net, ensuring adequate ventilation while maintaining the presence of the 30 houseflies in each unit. The mortality rate of the houseflies was observed and recorded.

To calculate the control effectiveness, the Henderson–Tilton [28] formula was employed. The calculation formula is as follows:

$$P_{2}=\left(1-\frac{I_{CK0}\times I_{PT1}}{I_{CK1\times I_{PT0}}}\right)\times 100$$

where

*CK0* represents the observation value in the control area before the experiment, *CK1* represents the observation value in the control area after the experiment, *PT0* represents the observation value in the treatment area before the experiment, and *PT1* represents the observation value in the treatment area after the experiment.

### 2.4. Statistical Analysis

Microsoft Excel 2016 was used to collate the experimental data, which are presented as mean ± standard error (SE). One-way analysis of variance (ANOVA) was performed using SPSS version 29.0 to compare differences among the groups. Tukey’s Honestly Significant Difference (HSD) test was subsequently used for post-hoc pairwise comparisons. Differences were considered statistically significant when *p* < 0.05 and nonsignificant when *p* > 0.05.

## 3. Results

### Preparation of M. brunneum WP

The optimal ratio of vacuum freeze-drying protectant to the spore suspension of *M. brunneum* was determined to be 1:1, based on its influence on the fungus’s germination rate and spore yield after freeze-drying (Table 1). Since UV light can negatively affect the germination and growth of *M. brunneum* WP, three chemical reagents were tested as potential UV protectants. Fluorescein sodium was ultimately selected as the UV protectant for *M. brunneum* WP. Different ratios of fluorescein sodium were tested, and the optimal concentration was found to be 3% (Table 2). Sodium dodecyl sulfate was selected as the wetting agent. By varying its concentration and measuring the wetting time, the optimal concentration was determined to be 3% (Table 3). Disodium methylene dinaphthalene sulfonate was used as a dispersant, and its suspension rate was tested at different concentrations. The optimal proportion was found to be 1% (Table 4). Based on these optimal ratios, *M. brunneum* WP was prepared, and its pH value and fineness were determined (Table 5). The pH and fineness of the prepared *M. brunneum* WP met the requirements for both application and the growth of *M. brunneum*(Table 6).

The combination of *M. brunneum* WP and low-dose deltamethrin prepared in this study effectively killed housefly larvae and pupae (Table 7). Seven days after application, the control efficacy of *M. brunneum* WP was 61.2%. After 3 days of treatment, deltamethrin’s control effect reached 71.1%, but this effect gradually declined over time. In contrast, the combined formulation of the two agents showed an increasing control effect as time progressed. On the 5th day, deltamethrin’s control effect was 62.1%, and by the 7th day, it had risen to 76.1%. The control efficacy of the combined formulation was superior to that of either agent used alone.

**Figure 2:**
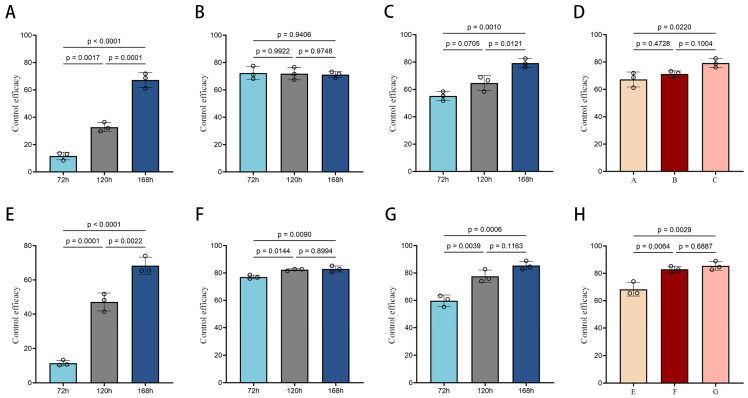
Control efficacy of *M. brunneum* WP, deltamethrin, and their combination against houseflies. (**A**) *M. brunneum* wettable powder (WP) control effect against housefly larvae and pupae; (**B**) Deltamethrin control effect against housefly larvae and pupae; (**C**) Combined *M. brunneum*–deltamethrin control effect against housefly larvae and pupae; (**D**) Comparative control effect of treatments on housefly larvae and pupae at 7 days. (**E**) *M. brunneum* WP control effect against adult houseflies; (**F**) Deltamethrin control effect against adult houseflies; (**G**) Combined *M. brunneum*–deltamethrin control effect against adult houseflies; (**H**) Comparative control effect of treatments on adult houseflies at 7 days. (The *p*-values in the figure were derived from Tukey HSD tests, and *p* < 0.05 denotes significant differences between groups).

Further analysis of the results (Figure 2A–D) demonstrated that the control effect of *M. brunneum* WP in combination with deltamethrin on housefly larvae and pupae significantly increased over time, while the effect of deltamethrin alone decreased. By day 7, the control effects of the three treatments at different stages of housefly development showed that the combination of *M. brunneum* WP and deltamethrin was more effective than the individual treatments. The combined formulation exhibited particularly strong control effects, and *M. brunneum* WP alone also showed a significant control effect on housefly larvae and pupae.

After treating adult houseflies with the insecticide, we observed that the mortality rate for deltamethrin was 73.3% after 1 day, but it remained relatively unchanged over time. In contrast, the mortality rate in the combined formulation group continued to increase, reaching 68.32% after 7 days of treatment with *M. brunneum* WP. As the treatment duration extended, the control effect of the combined formulation on adult houseflies gradually improved, reaching 85.42% at 7 days, which was the highest result among all treatments.

Further data analysis (Figure 2E–H) showed that the control effect of deltamethrin did not change significantly after the third day. At 7 days, the difference between the effects of *M. brunneum* WP and deltamethrin was highly significant, with both treatments exceeding 60%, indicating that *M. brunneum* WP has a strong control effect on houseflies. Although the difference between the combined formulation and deltamethrin was smaller, the combined formulation showed slightly better control at 7 days compared to deltamethrin alone. Statistical analysis confirmed that *M. brunneum* WP has a strong control effect on houseflies, and its efficacy is significantly enhanced when combined with deltamethrin.

## 4. Discussion

Globally, research and application of using entomopathogenic fungi for biological pest control have increasingly become an important component of sustainable agriculture [29,30,31,32,33]. The combined use of chemical pesticides and entomopathogenic fungi provides a sustainable strategy for livestock, reducing costs, resistance, toxicity, and management, while also decreasing residues in animal products and the environment [34,35,36,37,38]. However, several challenges exist in practical applications, especially when targeting the control of houseflies on pastures. One issue is the premature germination of conidia during transportation and storage, which affects their efficacy [39]. Published trials have shown that the efficacy of *M. brunneum* can be affected by varying temperature and humidity conditions, making it sensitive to environmental factors [40]. Another challenge is the increased labor costs associated with the simultaneous application of *M. brunneum* suspensions and chemical pesticides. Additionally, there is concern about potential non-target effects and ecological disruption. It should be emphasized that entomopathogenic fungi such as *Metarhizium brunneum* may have negative effects on non-target parasitoids and predators, which could disrupt the ecological balance in the studied area [41]. In the study by Ilya R Fischhoff et al., application of *M. brunneum* Met52 in an agricultural environment is unlikely to have significant negative effects on non-target arthropod populations or communities, while Johanna Mayerhofer et al. found that *M. brunneum* may not adversely affect soil microbial communities [42,43]. Studies by Federico Cappa et al. demonstrated that the entomopathogenic fungus *Beauveria bassiana* induces multiple adverse effects on *Polistes dominula* [44]. This ecological risk underscores the importance of prioritizing long-term impacts on natural enemy insect populations when promoting fungal insecticides, and calls for further evaluation of their environmental safety through field-based microcosm experiments.

Given these challenges, freeze-drying technology allows biological samples to be rapidly dehydrated under low temperatures and pressure, preserving their properties and making them suitable for preserving biocontrol agents like entomopathogenic fungi [45,46]. This study developed a freeze-dried formulation of *M. brunneum* and deltamethrin WP, providing a long-term storage and transportation solution that significantly reduces logistics costs and simplifies usage procedures.

Since *M. brunneum* conidia are susceptible to UV radiation during application, we initially screened three common UV protectants—skim milk, sodium ascorbate, and fluorescein sodium—for preparing *M. brunneum* wettable powder [47]. Tests demonstrated that fluorescein sodium had the least impact on *M. brunneum* germination and provided the best UV protection. Additionally, within the 1–5% range of fluorescein sodium concentration, UV protection capacity initially increased and then decreased, indicating that more is not always better. Other evaluation criteria for WPs include suspension rate, wetting time, and fineness. Based on these standards, dispersants and wetting agents were added to optimize the formulation, enhancing the performance of *M. brunneum* WP. Optimization of wetting agent ratios revealed that different proportions could vary wetting times by over 20 s. Dispersants improve the suspension stability of *M. brunneum* conidia, playing a crucial role in enhancing outdoor spraying efficacy.

Notably, most published trials indicate that the application dosage of *M. brunneum* ranges from 10^6^ to 10^8^ spores/mL, showing significant effectiveness against target pests [12,48,49]. In this study, a similar dosage (1 × 10^6^ conidia/mL) was used, and validated in preliminary laboratory tests for killing adult and larval houseflies. Variations in dosage can be attributed to differences in growth rates and predation efficiencies among different *M. brunneum* isolates, necessitating adjustments in dosing regimens.

Previous laboratory studies confirmed that *M. brunneum* strain KVL04-57 effectively infects and kills houseflies. After confirming the compatibility between *M. brunneum* and deltamethrin, we prepared a WP formulation. In the present work, we evaluated the efficacy of chemical compounds (deltamethrin), biological compounds (*M. brunneum*), and their combination against houseflies at different developmental stages. Field trial observations showed that the *M. brunneum* WP effectively infected and killed housefly larvae, pupae, and adults. Additionally, combining low doses of deltamethrin with *M. brunneum* resulted in higher mortality rates for both larvae and adults compared to using *M. brunneum* alone, increasing mortality rates by 11.98% and 17.1%, respectively. This approach reduced deltamethrin usage while enhancing the efficacy of *M. brunneum* in controlling houseflies. The combined use of *M. brunneum* with low-dose chemical pesticides produced synergistic effects, enhancing lethal efficacy against target pests. Moreover, the LT50 values for housefly larvae, pupae, and adults were shortened to varying degrees when *M. brunneum* was combined with low-dose deltamethrin.

These findings are in line with multiple previous studies. Yadav et al. (2019) observed that combining *M. brunneum* with low-dose chemical pesticides significantly improved control of *Maconellicoccus hirsutus*, with markedly reduced LT_50_ values [25]. Their work focused on a different pest species but still showed the positive impact of the combined approach. Similarly, Zottele et al. also found that the combination of *M. brunneum* with low-dose chemical pesticides effectively enhanced pest control in maize fields [15], highlighting the broad-spectrum applicability of such combinations in different agricultural settings. Sebastian A. Pelizza et al. investigated the control efficacy of the combined use of chemical insecticides and entomopathogenic fungi against the soybean pest Rachiplusia nu in Argentina. They found that the combined treatment resulted in a higher larval mortality rate than the use of single agents. For example, the combination of gamma-cyhalothrin and *Beauveria bassiana* (LPSc 1067) had the best effect, which is consistent with our study [38]. Although our study and Pelizza’s study used different fungi and targeted different pests, both reached the conclusion that the combination of chemical insecticides and entomopathogenic fungi is a promising pest control strategy. Pelizza’s research emphasized indicators like the in vitro viability of fungal conidia, while our study centered on the temporal changes in control efficacy.

Overall, the consistent positive results across these diverse studies further support the potential of combining chemical insecticides and entomopathogenic fungi as an effective and sustainable pest control strategy. However, the specific effectiveness can vary depending on factors such as the pest species, the choice of chemical and biological agents, and the environmental conditions. Future research should focus on optimizing these combinations for different pests and agricultural systems.

## 5. Conclusions

The results demonstrate that the prepared *M. brunneum* WP (WP) exhibited good efficacy against housefly larvae, pupae, and adult flies. After 7 days of treatment, the control rates for housefly larvae and housefly adults were 67.22% and 68.32%, respectively. When low-dose deltamethrin was combined with *M. brunneum*, the control rates for housefly larvae and housefly adults increased to 79.20% and 85.42%, respectively. These findings indicate that the formulation is effective for controlling ectoparasites in livestock and poultry.

Moreover, the combination of low-dose deltamethrin and *M. brunneum* WP not only enhances the control efficacy but also reduces the required amount of deltamethrin, making it a more environmentally friendly and economically viable option. This study provides valuable insights into the development of integrated pest management strategies for controlling houseflies in agricultural settings.

## Figures and Tables

**Table 1:** Screening of lyoprotectant ratios.

Lyoprotectant/Fungus	Spore Germination Rate (%)
1:1	38.67 ± 2.05 a
1:2	27.33 ± 2.62 b
1:3	16.33 ± 1.70 c
NS	15.67 ± 3.30 c

Perform a difference comparison between the control group and the high-, medium-, and low-dose groups. Different letters within the same column indicate significant differences (*p* < 0.05), while the same letters indicate no significant differences (*p* > 0.05).

**Table 2:** Optimization of UV protectant ratio.

Ratio of Sodium Fluorescein	Spore Germination Rate After 60 s of UV Irradiation
1%	13.33 ± 4.78 a
2%	14.33 ± 1.25 a
3%	34.33 ± 7.04 c
4%	8.00 ± 2.45 d
5%	12.33 ± 2.05 a

Perform a difference comparison between the control group and the high-, medium-, and low-dose groups. Different letters within the same column indicate significant differences (*p* < 0.05), while the same letters indicate nonsignificant differences (*p* > 0.05).

**Table 3:** Effect of wetting agent proportion on wetting time.

Wetting Agent Ratio	Wetting Time (s)
1%	42.40 ± 0.57 a
2%	25.02 ± 1.19 c
3%	19.70 ± 1.24 e
4%	36.04 ± 0.49 f
5%	43.72 ± 1.94 a

Perform a difference comparison between the control group and the high-, medium-, and low-dose groups. Different letters within the same column indicate significant differences (*p* < 0.05), while the same letters indicate nonsignificant differences (*p* > 0.05).

**Table 4:** Effect of dispersant ratio on suspension rate.

Dispersant Ratio	Suspension Rate (%)
1%	94.97 ± 0.13 a
2%	91.83 ± 0.50 c
3%	90.73 ± 0.58 c
4%	93.23 ± 0.34 b
5%	91.57 ± 0.39 c

Perform a difference comparison between the control group and the high-, medium-, and low-dose groups. Different letters within the same column indicate significant differences (*p* < 0.05), while the same letters indicate nonsignificant differences (*p* > 0.05).

**Table 5:** Formulation components and composition of *M. brunneum* WP.

Formula	Components	Ratios
Freeze-dried chlamydospore powder	*M. brunneum* freeze-dried powder	20%
UV protectants	sodium fluorescein	3%
Moistener	Sodium dodecyl sulfate	3%
Dispersant	Disodium methylenebisnaphthalenesulphonate	1%
Carrier	Diatomite	Top-up to 100%

**Table 6:** Quality test results of *M. brunneum* WP.

Quality Metric	GB Standard	Measured Value
Spore Content (1 × 10^6^ spores/g)	≥50%	74.17 ± 6.33%
Wetting Time	≤120 s	67.21 ± 1.34 s
Suspension Rate	≥70%	81.30 ± 4.16%
Moisture Content	≤8%	1.20 ± 0.22%
Carrier	Diatomite	Top-up to 100%
Fineness	≥90%	98.60 ± 0.26%
pH	6–8	6.98 ± 0.06
Storage Stability	≥90 d	120-day stability

**Table 7:** Control efficacy of *M. brunneum* WP against housefly larvae and adults in farm settings.

Groups		Control Efficacy (%)		
	Treatment Group	72 h	120 h	168 h
Housefly larvae	A	11.62 ± 1.60	32.73 ± 1.94	67.22 ± 3.15
	B	72.29 ± 2.75	71.90 ± 2.60	71.19 ± 1.33
	C	55.17 ± 1.91	64.64 ± 3.17	79.20 ± 1.88
Housefly	A	11.46 ± 0.94	47.08 ± 3.04	68.32 ± 2.87
	B	77.03 ± 0.80	82.33 ± 0.43	82.90 ± 1.27
	C	59.74 ± 2.41	77.56 ± 2.65	85.42 ± 1.82

## Data Availability

The raw data supporting the conclusions of this article will be made available by the authors upon request.

## Abbreviations

The following abbreviations are used in this manuscript:

WP	Wettable powder
PDA	Potato dextrose agar
DM	Deltamethrin
EPF	Entomophagous fungus
*M. brunneum*	*Metarhizium brunneum*
UV	Ultraviolet
